# Safety and efficiency of repeat salvage lymph node dissection for recurrence of prostate cancer using PSMA-radioguided surgery (RGS) after prior salvage lymph node dissection with or without initial RGS support

**DOI:** 10.1007/s00345-023-04534-5

**Published:** 2023-07-29

**Authors:** Fabian Falkenbach, Sophie Knipper, Daniel Koehler, Francesca Ambrosini, Thomas Steuber, Markus Graefen, Lars Budäus, Matthias Eiber, Lukas Lunger, Flemming Lischewski, Matthias M. Heck, Tobias Maurer

**Affiliations:** 1 Martini-Klinik Prostate Cancer Center Hamburg-Eppendorf, Martinistr. 52, 20246 Hamburg, Germany; 2 Department of Diagnostic and Interventional Radiology and Nuclear Medicine, University Medical Center Hamburg-Eppendorf, Hamburg, Germany; 3 Department of Urology, IRCCS Policlinico San Martino Hospital, University of Genova, Genoa, Italy; 4 Department of Urology, University Medical Center Hamburg-Eppendorf, Hamburg, Germany; 5 Department of Nuclear Medicine, Rechts der Isar Medical Center, Technical University of Munich, Munich, Germany; 6 Department of Urology, Rechts der Isar Medical Center, Technical University of Munich, Munich, Germany

**Keywords:** Hormone-sensitive, PET, Positron emission tomography, Metastasis directed therapy, Pelvic recurrence

## Abstract

**Background and objective:**

Metastasis-directed therapy is a feasible option for low PSA, recurrent locoregional metastatic prostate cancer. After initial salvage surgery, patients with good response might consider a repeat salvage surgery in case of recurrent, isolated, and PSMA-positive metastases. This analysis aimed to evaluate the oncological outcome and safety of repeat PSMA-targeted radioguided surgery (RGS) after either prior RGS or “standard” salvage lymph node dissection (SLND).

**Materials and methods:**

We identified 37 patients undergoing repeat RGS after prior SLND (*n* = 21) (SLND-RGS) or prior RGS (*n* = 16) (RGS-RGS) between 2014 and 2021 after initial radical prostatectomy with or without pelvic radiation therapy at two German tertiary referral centers. Kaplan–Meier analyses and uni-/multivariable Cox regression models were used to investigate factors associated with biochemical recurrence-free survival (BRFS) and treatment-free survival (TFS) after repeat salvage surgery.

**Results and limitations:**

Complete Biochemical Response (cBR, PSA < 0.2 ng/ml) was observed in 20/32 patients (5 NA). Median overall BRFS [95% confidence interval (CI)] after repeat salvage surgery was 10.8 months (mo) (5.3–22). On multivariable regression, only age (HR 1.09, 95% CI 1.01–1.17) and preoperative PSA (HR 1.23, 95% CI 1.01–1.50) were associated with shorter BRFS, although PSA (HR 1.16, 95% CI 0.99–1.36) did not achieve significant predictor status in univariable analysis before (*p* value = 0.07). Overall, one year after second salvage surgery, 89% of the patients (number at risk: 19) did not receive additional treatment and median TFS was not reached. Clavien–Dindo grade > 3a complications were observed in 8% (3/37 patients). Limitations are the retrospective evaluation, heterogeneous SLND procedures, lack of long-term follow-up data, and small cohort size.

**Conclusion:**

In this study, repeat RGS was safe and provided clinically meaningful biochemical recurrence- and treatment-free intervals for selected cases. Patients having low preoperative PSA seemed to benefit most of repeat RGS, irrespective of prior SLND or RGS or the time from initial RP/first salvage surgery.

**Supplementary Information:**

The online version contains supplementary material available at 10.1007/s00345-023-04534-5.

## Introduction

The management of biochemical failure after curative intent primary treatment with the detection of oligo-metastatic recurrence of prostate cancer (PCa) remains challenging. Despite a shortage of prospective studies, emerging data support the role of metastasis-targeted therapies (MDT). By this, initiation of systemic treatment may be postponed in selected cases, either by surgery or radiation [1–3]. However, delayed or immediate androgen deprivation therapy (ADT) often constitutes the standard of care for these patients [4]. Salvage lymph node dissection (SLND) for nodal recurrence can achieve a complete biochemical response (cBR, defined as postoperative PSA of 0.2 ng/ml or below) in 28% [5] up to 46% [6] and prolong ADT-free survival compared to expectant management alone [2, 7].

In a small case series, repeat SLND achieved cBR in 4 out of 10 patients, and the median time to PSA relapse to the preoperative value was 23 months for repeat SLND [8]. Accordingly, MDT, even in a repeat setting, might improve cancer-specific survival compared with the standard of care for nodal, oligo-recurrent prostate cancer [9]. In a long-term follow-up, MDT might even be curative in a very small proportion of patients, while most patients are likely to require a multimodal approach [10, 11].

Prostate-specific membrane antigen (PSMA) positron emission tomography (PET) has led to the detection of small, localized recurrence of prostate cancer at low PSA values [12, 13]. Especially, these patients seem to benefit most from MDT. In a systematic review from 2019, the 2 and 5 years biochemical progression-free survival rates after SLND with prior PSMA PET imaging ranged from 23 to 64% and from 6 to 31%, respectively [14]. Salvage Surgery using RGS increases cancer extraction yield by extensive in vivo and ex vivo measurements and immediate feedback for the surgeon [15–17], while providing a reasonable safety profile [18]. The RGS approach offers hereby the precision needed for the definitive treatment of small nodal recurrence [17] and therefore became a routine procedure in open and even robotic surgery [19] at our institutions. In selected cases with a low preoperative PSA value and a single lesion, RGS can achieve cBR in up to 84% [20].

However, the question remains if it is reasonable to offer selected patients a repeat salvage RGS after failure of initial SLND or RGS. We therefore aimed to evaluate the oncological outcome and safety of repeat PSMA-RGS after either prior RGS or “standard” SLND.

## Patients and methods

### Patients

Within the prospectively collected clinical database of two tertiary care centers with 524 RGS procedures, we identified 37 patients, who received a repeat RGS for oligo-metastatic recurrence of hormone sensitive prostate cancer between 2014 and 2021 after prior SLND or RGS after initial radical prostatectomy with or without pelvic radiation therapy. All patients received PSMA PET imaging prior referral for surgery. We excluded two patients with repeat RGS performed elsewhere (without documentation) and two patients with atypical metastasectomy (cervical, visceral). All patients were informed about the experimental nature of PSMA-radioguided SLND and provided informed consent for the procedure and data analysis. This retrospective analysis was approved by the institutional review boards of Hamburg (2019-PS-09; PV7316) and Munich (number 336/18 S), Germany. Questionnaires were used for the follow-up. All data were prospectively stored in an institutional database (FileMaker Pro 10; FileMaker Inc., Santa Clara, CA, USA).

### Procedure of (repeat) salvage surgery using RGS

Repeat RGS procedures were performed as reported for initial RGS procedures [16, 19, 21, 22]. Radioguidance was achieved through extensive in vivo and ex vivo measurements using a gamma probe (Crystal Probe CXS-SG603 or DROP-IN gamma probe; Crystal Photonics, Berlin, Germany) with acoustic and numerical feedback as a response to ^99m^Tc radioactivity. Further excision/search is prompted in cases of insufficient ex vivo signals [16]. All RGS procedures were performed by experienced, high-volume surgeons.

### Outcomes of interest

Biochemical recurrence-free survival (BRFS, defined as postoperative PSA < 0.2 ng/ml without further treatment) and treatment-free survival (TFS, defined as survival without further therapy) as well as in comparison to the BRFS after initial SLND / RGS and after RP were evaluated. Furthermore, the rate of cBR (defined as PSA < 0.2 ng/ml) without additional treatment was determined 2–16 weeks following repeat salvage surgery. The time between the first and second salvage surgeries and the time between RP and first salvage surgery were measured. Postoperative complications were classified according to Clavien–Dindo [23].

### Statistical analyses

Descriptive statistics included frequencies and proportions for categorical variables. The medians and interquartile ranges (IQR) were reported for continuously coded variables. The statistical significance of differences in medians and proportions was evaluated using the Kruskal–Wallis and Chi-square tests, respectively. For comparison of contingency tables, the Fisher’s exact test was applied. Kaplan–Meier plots graphically depict the BRFS and cBR after repeat salvage surgery. Univariable and multivariable Cox regression models were used to investigate the association between oncological outcomes (BRFS, cBR, TFS) and selected variables [age at repeat surgery (continuously coded), Gleason Grade Group at RP (I–II vs. III–V), radiation therapy post RP (yes vs. no), time between initial SLND/RGS and repeat RGS (continuously coded), PSA at repeat RGS (continuously coded), number of PSMA PET-positive lesions prior to repeat RGS (continuously coded), and localization of PSMA PET-positive lesions (pelvic vs. retroperitoneal and pelvic vs. retroperitoneal only)]. All tests were two sided, with the significance level set at *p* < 0.05. The R software environment for statistical computing and graphics (version 3.4.3, R Foundation for Statistical Computing) was used for all statistical analyses.

## Results

### Patients’ characteristics and comparison of groups

Of the 37 patients who underwent repeat salvage surgery using RGS after prior SLND (*n* = 21) or RGS (*n* = 16), all received RP as primary treatment. Baseline characteristics at RP were similar among both groups with a tendency for more severe disease in the RGS-RGS cohort (supplementary Table 2). Of note, only 3/16 (RGS-RGS) and 4/21 (SLND-RGS) of patients (equals 19% in both groups) were initially lymph node positive at RP. Initial lymph node dissection at RP was performed in 33 of 37 patients (89%).

At initial salvage surgery, specimens removed were without histological proof of cancerous tissue in 1 of 16 patients (6%) undergoing RGS and 6 of 21 patients (29%) undergoing SLND. The median age at repeat salvage surgery was 70 years (IQR: 62–74 years, RGS–RGS) or 69 years (IQR: 65–70 years, SLND-RGS). Patients after SLND had a higher median PSA before repeat salvage surgery (RGS-RGS: 0.66 ng/ml vs. SLND-RGS: 1.16 ng/ml), while the number of pathologically positive lesions was comparable (*p* = 0.37). All patients showed 1–3 metastatic soft tissue lesions, as determined by PSMA PET imaging (Table 1).

**Table 1:** Baseline characteristics at initial and repeat salvage surgery of the 37 patients included in this study

	RGS-RGS, (*n* = 16)		SLND-RGS (*n* = 21)	
	1. RGS	2. RGS	1. SLND	2. RGS
Time between RP and salvage surgery, months, medians (IQR)	58 (44, 111)	90 (70, 126)	63 (24, 96)	88 (50, 141)
Time between first and repeat salvage surgery, months, medians (IQR)	28 (18, 36)		25 (10, 41)	
Age at salvage surgery, median (IQR)	68 (60, 72)	70 (62, 74)	65 (63, 68)	69 (65, 70)
PSA prior to salvage surgery, ng/ml, median (IQR)	0.84 (0.35, 2.03)	0.66 (0.42, 1.31)	0.50 (0.28, 2.09)	1.16 (0.89, 2.25)
PSMA PET avid lesions prior salvage, *n* (%)				
1	12 (75%)	9 (56%)	3 (14%)	13 (62%)
2	2 (13%)	6 (38%)	0	7 (33%)
3	2 (13%)	1 (6%)	0	1 (5%)
NA	0	0	18 (86%)	0
Total number of avid lesions treated per cohort	22	22		21
PSMA PET localization, *n* (%)^a^				
Pelvic	14 (88%)	11 (69%)	3 (14%)	19 (91%)
Retroperitoneal	2 (6%)	5 (31%)	0	2 (10%)
NA	0	0	18 (86%)	0
Removed metastasis, *n* (%)				
No cancer removed	1 (6%)	2 (13%)	6 (29%)	0
1	8 (50%)	6 (38%)	3 (14%)	11 (52%)
2	3 (19%)	5 (31%)	3 (14%)	5 (24%)
3	2 (13%)	3 (19%)	0	1 (5%)
≥ 4	2 (13%)	0	0 NA: 9 (43%)	4 (19%)

*RGS* PSMA-targeted radioguided surgery, *SLND* salvage lymph node dissection, *RP* radical prostatectomy, *PSA* prostate-specific antigen, *NA* not assigned, *RT* radiotherapy, *IQR* interquartile range

^a^The sums of pelvic and retroperitoneal PET location may exceed the number of patients due to metastasis on both locations

### Oncological outcomes

**Fig. 1:**
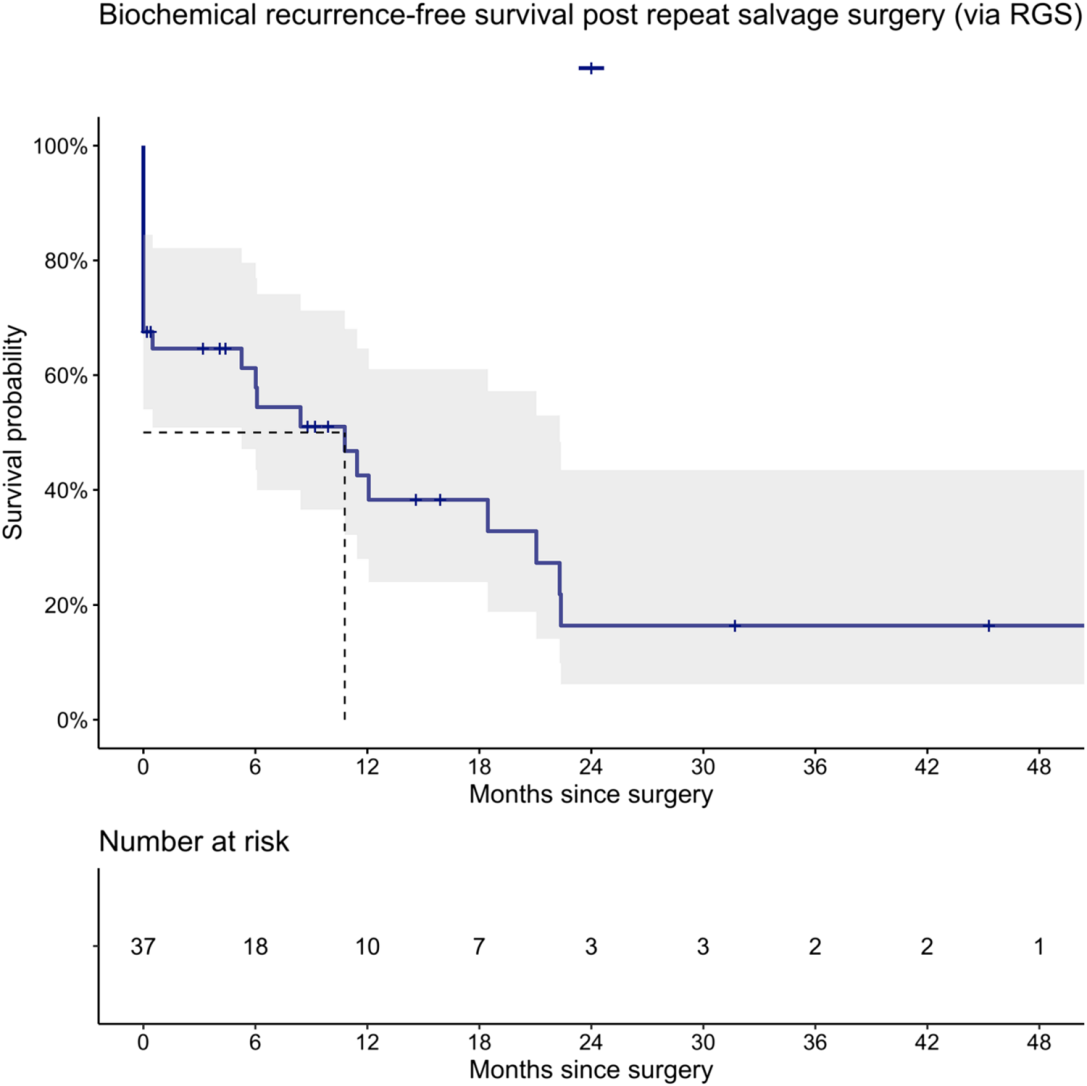
Kaplan–Meier analyses depicting biochemical recurrence-free survival rates in 37 patients treated with repeat PSMA-RGS after initial SLND or prior PSMA-RGS

At 2–16 weeks after repeat salvage surgery, 20/32 patients (63%, 5 NA) achieved cBR. The median (IQR) follow-up for patients who did not experience biochemical recurrence was 9.0 (3.9–12.3) months. The median (IQR) follow-up for patients who did not receive further therapy was 10.8 (5.7–25.0) months. One year after repeat salvage surgery, 43% of the patients (number at risk: 10) did not experience biochemical recurrence. One year after repeat salvage surgery, 89% of the patients (number at risk: 19, supplementary Fig. 3) did not receive additional treatment. The median overall BRFS (95%-CI) after repeat salvage surgery was 10.8 months (5.3–22.3, Fig. 1) and the median TFS was not reached (supplementary Figs. 3 and 4). None of the patients died during follow-up.

In univariable Cox regression analyses, age (HR 1.06, 95% CI 1.00–1.12), pN1 at RP (HR 2.72, 95% CI 1.03–7.18), number of lesions on PSMA PET (HR 2.14, 95% CI 1.1–4.17), and combined pelvic/retroperitoneal localization (HR 3.93, 95% CI 1.05–14.71) were significantly associated with shorter BRFS in the overall population (*p* < 0.05, supplementary Table 3). Although preoperative PSA (HR 1.16, 95% CI 0.99–1.36) did not achieve significant predictor status in univariable analysis (*p* = 0.07), it was included in multivariable analysis because of small sample size and our experiences in earlier studies [24].

In multivariable Cox regression analyses, only age (HR 1.09, 95% CI 1.01–1.17) and preoperative PSA (HR 1.23, 95% CI 1.01–1.50) were independently associated with shorter BRFS (*p* < 0.05).

### Patient safety

The rate of major complications (Clavien–Dindo grade ≥ 3) within the entire cohort was 3/37 patients (8%). Of those, 2/3 cases were in patients who had previous radiation therapy. All major complications were due to bowel injury (Supplementary Table 4). These were treated in one case by simple suture (for minor rectal injury) and in two cases by the creation of an enterostomy (for sigma perforation). Interestingly, these complications were all reported in the SLND-RGS group. There were no major complications at RGS after prior RGS. Given the small sample size these differences are not statistically significant (*p*  = 0.09).

## Discussion

To our knowledge, this is the first study on repeat salvage surgery for oligo-recurrent metastatic prostate cancer using the enhanced capabilities of RGS. Here, patients can be generally classified into two subgroups: those with insufficient initial salvage surgery and those with repeated recurrence after successful initial salvage surgery. In the first subgroup, RGS confirmed its higher tumor removal rate, especially after prior template dissection (in all patients in this group cancerous tissue was removed). As stated before, data on repeat salvage surgery are limited; therefore, oncological comparison of the RGS vs. SLND for repeat salvage surgery is not possible. In a case series of ten patients, positive lymph nodes were removed in 90% and a cBR was observed in 40% at repeat SLND [8]. Within our cohort using RGS, we achieved a positive retrieval rate of 95% and cBR of 63%. The retrieval rate of repeat RGS was also consistent with the initial RGS in this study (94%). Interestingly, the only two cases of repeat RGS without cancer removal were observed in patient with prior RGS. In conclusion, the advantages of RGS in the repeat setting are similar to the initial salvage surgery situation [15].

Concerning the proposed subgroup of patients with sufficient initial salvage surgery and true second oligo-recurrence, it is worth comparing repeat with initial salvage surgery. Still, even initial SLND itself is considered an experimental therapy. In our own cohort, as previously reported [22], cBR was observed in 78% of all patients receiving initial RGS, compared to 63% in repeat RGS. Although this difference can partly be explained by the worse baseline characteristics and potential negative selection bias of our repeat salvage surgery group, the TFS at 1 year of follow-up was similar (88% for initial vs. 89% for repeat RGS). The efficiency of RGS in the repeat setting was further confirmed by our cBR rates in the repeat setting as compared with those of (initial) SLND. In a systematic review, cBR rates ranged from 13 to 80% (mean 44%) in comparison to our 63% for the repeat setting, while TFS at 1 year was not reported [14]. Furthermore, the influence of treatment timing within metastasis-directed therapy has not been sufficiently analyzed. Slow PSA kinetics at BCR, which may be found as a surrogate parameter for slow development of new lesions, has a preferable risk profile [25]. The shorter median time between RP and first salvage (61 months, IQR: 32–102 months) vs. first salvage and second salvage surgery (28 months, IQR: 14–41 months) may indicate increasing aggressivity within this pre-selected cohort (*p*  = 0.26), although validity of this statement may be limited due to adjuvant radiation therapy after RP in half of the patients. Consequently, the shorter time for BRFS for repeat RGS is plausible. To summarize, while it remains partly controversial, if MDT prolongs time to initiation of systemic treatment or castration-resistance or might even cure oligo-recurrent PCa [2], repeat salvage SLND using RGS shows similar efficacy as in the initial salvage situation and thus may potentially be offered patients with the same limitations as in the initial setting.

In view of the unproven oncological benefit of repeat RGS, patient safety is, however, of utmost importance. The rate of major complications (Clavien–Dindo score ≥ 3) was 8%. Given our small sample size, this rate is similar to our previously reported major complication rate of 7% following RGS [24]. In comparison, in a systematic review by Ploussard et al., the rate of major complications for (initial) SLND varied among all studies between 0 and 20% (grade IIIa) and 16% (grade IIIb) [14]. In a pooled analysis, major complications occurred in 9% of SLND [26]. Although data for repeat salvage surgery remain limited, Grabbert et al. reported an overall major complication rate of at least 22% in a small case series [8]. Nevertheless, the high rate of bowel injury is disturbing and may be caused by two prior surgeries in the region.

However, the main key for initial as well as repeat RGS remains patient selection, as this is not a “*one-size-fits-all*” approach. On multivariable analysis, only age and PSA prior repeat RGS were significantly associated with shorter BRFS. A higher preoperative PSA value (often with a proposed cut-off of 4 ng/ml) has been correlated with worse outcomes after SLND [7, 11, 27, 28]. In accordance therewith, PSA at therapy has been reported as predictive factor for salvage treatment in general [29]. Also, localization and number of PET-positive lesions have been described predictive factors for salvage treatment [3, 24, 29] and were confirmed as such here. In contrast, Gleason grade group at RP and adjuvant radiotherapy did not reach statistical significance as also previously described by Suardi et al*.* [27]. Interestingly enough and contrary to our initial belief, the time between initial and repeat salvage surgeries did not translate in different outcomes. Although patients with RGS after SLND had a higher PSA at second RGS and the time between first and second salvage surgeries was shorter, patients in both groups had similar rates of PSMA PET-positive lesions and pathologically proven metastasis resected at the second RGS. This is also remarkable as the RGS-RGS group had more adverse pathology at initial RP.

Nevertheless, the results of our analysis must be interpreted with caution. First, a control group is lacking. Such a group might consist of patients undergoing watchful waiting/active surveillance, radiotherapy, and/or systematic treatment regimens. Randomized prospective studies in this field of targeted surgical treatment are highly desired. Most of our patients presented asymptomatic with a low PSA in an oligo-recurrent setting. Thus, those patients do not face imminent life-threatening risks, but the side effects of continuous systemic therapy once initiated upon further progression. Recently, long-term outcomes of SLND were reported to be somewhat disappointing [10]. High-level evidence for SLND and even more so for repeat SLND is missing [14], while phase II trials like ORIOLE demonstrated less progression under MDT vs. expectant management for radiation therapy [2, 30]. However, follow-up was too short and the cohort size too small to evaluate clinically significant endpoints. Also in our study, cBR and TFS without standardized trigger for next treatment are weak surrogate endpoints. Furthermore, the effect of selection bias on many different layers should not be underestimated. Finally, the small sample size limits the statistical validity and especially the multivariable analysis with six variables is prone to overfitting, which may introduce bias. However, the applied statistical tests and modeling were primarily calculated for exploratory purposes to compare both groups. Furthermore, a larger sample size is difficult to obtain in this setting of repeat SLND as it represents a procedure for highly selected patients treated only at expert centers. Therefore, this sample is still extremely valuable.

## Conclusion

In selected cases, repeat RGS might delay the need for systemic treatment, while offering a reasonable safety profile. The effectiveness is independent of time from initial RP or first salvage surgery, adjuvant radiotherapy after RP, or the kind of initial salvage surgery (RGS/ SLND). Limitations are a missing control group, the retrospective evaluation, the small cohort, and lack of long follow-up data.

## Supplementary Information

Below is the link to the electronic supplementary material.

Supplementary file1 (DOCX 22512 KB)
