# Supplementary material for: Safety and efficiency of repeat salvage lymph node dissection for recurrence of prostate cancer using PSMA-radioguided surgery (RGS) after prior salvage lymph node dissection with or without initial RGS support
Source: World J Urol. 2023 Jul 29;41(9):2343–50. doi: 10.1007/s00345-023-04534-5 (PMC10465644; doi:10.1007/s00345-023-04534-5)
Supplement: Supplementary file 1 — Supplementary file1 (DOCX 22512 KB) [file 345_2023_4534_MOESM1_ESM.docx]

Supplementary Data

|  | **RGS-RGS, (n=16)** | **SLND-RGS (n=21)** | **p value*** |
| --- | --- | --- | --- |
| **Year of initial RP**, Median (IQR) | 2013 (2008, 2014) | 2010 (2006, 2012) | 0.24 |
| **PSA at RP**, ng/ml, Median (IQR) | 9.4 (5.8, 16.0) | 9.0 (5.8, 13.8) | 0.92 |
| **pT stage at RP,** n (%) |  |  | 0.35 |
| pT2 | 5 (31%) | 9 (43%) |  |
| pT3a | 7 (44%) | 8 (38%) |  |
| pT3b | 4 (25%) | 2 (10%) |  |
| NA | 0 | 2 (10%) |  |
| **Gleason grade group**, n (%) |  |  | 0.44 |
| I | 0 | 1 (5%) |  |
| II | 5 (31%) | 10 (48%) |  |
| III | 7 (44%) | 6 (29%) |  |
| IV | 1 (6%) | 2 (10%) |  |
| V | 2 (13%) | 0 |  |
| NA | 1 (6%) | 2 (10%) |  |
| **pN stage at RP**, n (%) |  |  | 0.32 |
| pN0 | 13 (81%) | 13 (62%) |  |
| pN1 | 3 (19%) | 4 (19%) |  |
| pNX / NA | 0 | 4 (19%) |  |
| **Lymph node yield at RP**, Median (IQR) | 13 (11, 16) | 7 (5, 15) | 0.21 |
| **No. of positive lymph nodes** at RP, n (%) |  |  | 0.64 |
| 1 | 2 (13%) | 1 (5%) |  |
| 2 | 1 (6%) | 2 (10%) |  |
| 3 (maximum) | 0 | 1 (5%) |  |
| **Surgical margin status**, n (%) |  |  | 0.44 |
| R0 | 14 (88%) | 14 (67%) |  |
| R1 | 1 (6%) | 3 (14%) |  |
| RX | 1 (6%) | 4 (19%) |  |
| **RT post RP**, n (%) |  |  | 0.70 |
| no RT | 8 (50%) | 8 (38%) |  |
| RT post RP | 8 (50%) | 13 (62%) |  |

Table 2: Baseline characteristics at initial radical prostatectomy (RP) of the 37 patients included in this study. All patients were treated with two salvage surgeries between 2014 and 2021 for biochemical recurrence after radical prostatectomy (RP) with positive lesions at prostate specific membrane antigen (PSMA)-positron emission tomography (PET) imaging.

RGS = PSMA-targeted radioguided surgery, SLND = salvage lymph node dissection, RP = radical prostatectomy, PSA= prostate specific antigen, NA= not assigned, RT= radiotherapy, IQR= interquartile range. * P values of the Chi-square test refer to the comparison of both groups.

|  | **Univariable Cox regression model** | | | | **Multivariable Cox regression model** | | | |
| --- | --- | --- | --- | --- | --- | --- | --- | --- |
| **Variables** | HR | CI 5% | CI 95% | p-value | HR | CI 5% | CI95% | p-value |
| Age at 2^nd^ salvage surgery (continuous)* | 1.06 | 1.00 | 1.12 | 0.046 | 1.09 | 1.01 | 1.17 | 0.03 |
| Gleason Grade Group at RP |  |  |  |  |  |  |  |  |
| *I-II* | *Ref.* |  |  |  |  |  |  |  |
| *III-V* | 1.5 | 0.63 | 3.6 | 0.4 |  |  |  |  |
| pN stage at RP* |  |  |  |  |  |  |  |  |
| *pN0/x* | *Ref.* |  |  |  |  |  |  |  |
| *pN1* | 2.72 | 1.03 | 7.18 | 0.04 | 2.06 | 0.68 | 6.23 | 0.2 |
| RT post RP |  |  |  |  |  |  |  |  |
| *No* | *Ref.* |  |  |  |  |  |  |  |
| *Yes* | 1.39 | 0.60 | 3.19 | 0.4 |  |  |  |  |
| 2^nd^ RGS after |  |  |  |  |  |  |  |  |
| *RGS* | *Ref.* |  |  |  |  |  |  |  |
| *sLND* | 0.79 | 0.34 | 1.84 | 0.6 |  |  |  |  |
| Time initial to repeat salvage surgery (continuous), in years | 1.06 | 0.91 | 1.23 | 0.5 |  |  |  |  |
| Time RP to PSMA-RGS (continuous), in years | 1.04 | 0.96 | 1.13 | 0.4 |  |  |  |  |
| PSA prior to repeat PSMA-RGS (continuous), ng/ml* | 1.16 | 0.99 | 1.36 | 0.07 | 1.23 | 1.01 | 1.50 | 0.04 |
| No. of PSMA PET positive lesions (continuous)* | 2.14 | 1.1 | 4.17 | 0.03 | 1.33 | 0.57 | 3.12 | 0.5 |
| Localization of PSMA PET positive lesions* |  |  |  |  |  |  |  |  |
| *pelvic only* | *Ref.* |  |  |  |  |  |  |  |
| *pelvic and retroperitoneal* | 3.93 | 1.05 | 14.71 | 0.04 | 2.37 | 0.55 | 10.3 | 0.3 |
| *retroperitoneal only* | 2.4 | 0.54 | 10.8 | 0.3 | 2.38 | 0.47 | 12.0 | 0.3 |

Table 3: Uni- and multivariable Cox regression models predicting biochemical-recurrence free survival after the repeat salvage surgery (n=37).

HR= Hazard ratio, CI= confidence interval, RP= radical prostatectomy, RT= radiotherapy, PSMA-RGS= prostate-specific membrane antigen radio-guided surgery, PSA= prostate-specific antigen, sLND= salvage lymph node dissection, PET= positron emission tomography.

** We included PSA prior to PSMA-RGS in the multivariate analysis despite the borderline p-value in the univariate analysis because this parameter had a significant predictor status in earlier studies.*

| **Complication Grade,** n (%) | **RGS-RGS, (n=16)** | **SLND-RGS (n=21)** | **All (n=37)** |
| --- | --- | --- | --- |
| none | 8 (50%) | 11 (52%) | 19 (51%) |
| I | 7 (44%) | 6 (29%) | 13 (35%) |
| II | 1 (6%) | 1 (5%) | 2 (5%) |
| III* | 0 | 2 (10%) | 2 (5%) |
| IV* | 0 | 1 (5%) | 1 (3%) |
| **Specific** **complication**, n (%) |  |  |  |
| Voiding issues | 2 (12%) | 2 (10%) | 4 (11%) |
| Bowel injury | 0 | 4 (19%) | 4 (11%) |
| Fever | 1 (6%) | 0 | 1 (3%) |
| Lymphocele | 0 | 1 (5%) | 1 (3%) |
| Diarrhoe | 1 (6%) | 0 | 1 (3%) |
| Obstipation | 2 (13%) | 1 (5%) | 3 (8%) |
| Peripheral neuropathy | 0 | 2 (10%) | 2 (5%) |
| Haematuria | 1 (6%) | 0 | 1 (3%) |
| Wound dehiscence | 1 (6%) | 0 | 1 (3%) |

Table 4: Complications of repeat salvage surgery using PSMA-RGS approach. Grading according to Clavien-Dindo. * All Complications ≥ III° were due to bowel injury (2 patients treated with the creation of enterostoma, 1 patient treated with rectal suture).


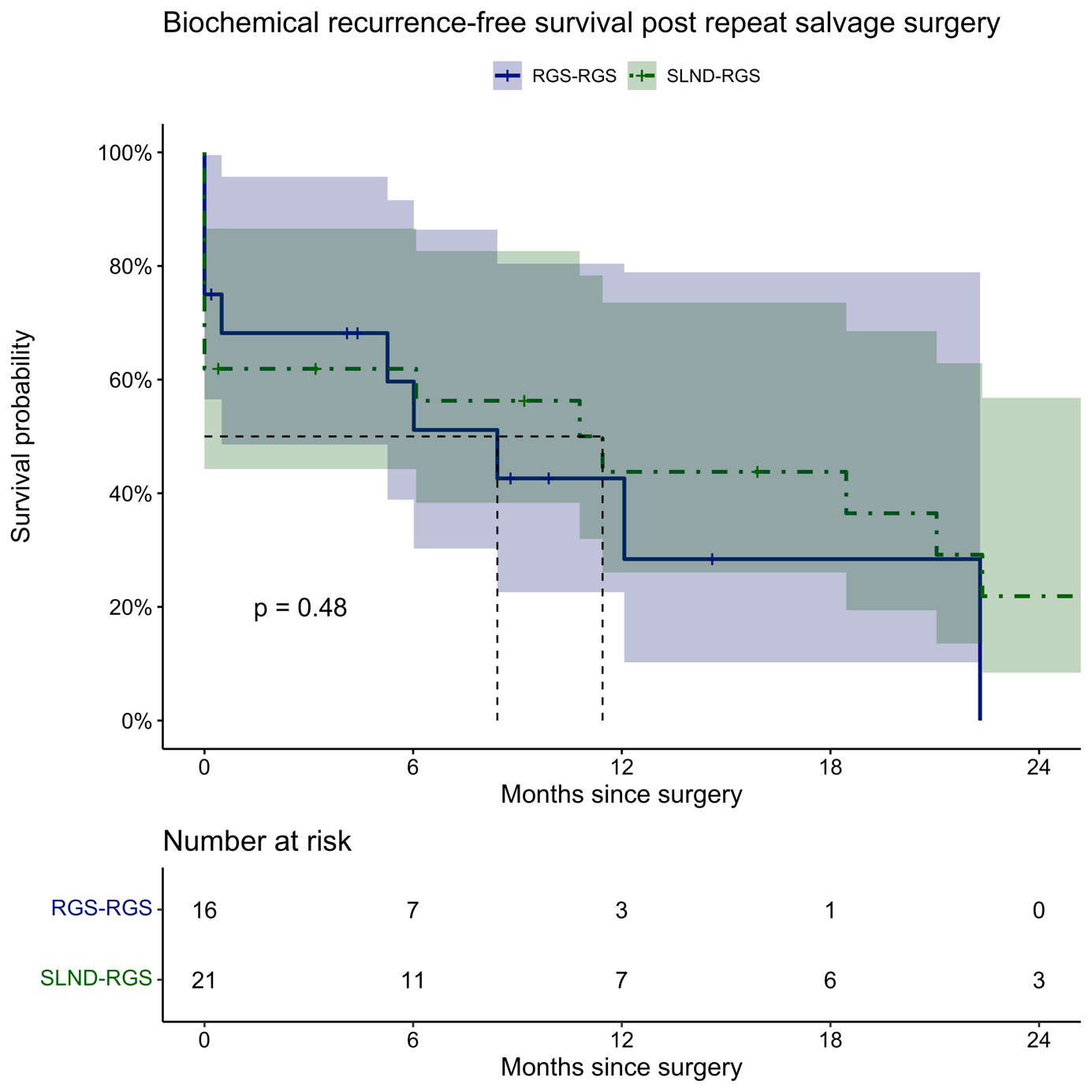


Fig. 2: Kaplan-Meier analyses depicting biochemical recurrence–free survival rates in patients treated with repeat PSMA–RGS divided by initial treatment (SLND-RGS in 21 pat. vs. RGS-RGS in 16 pat.).


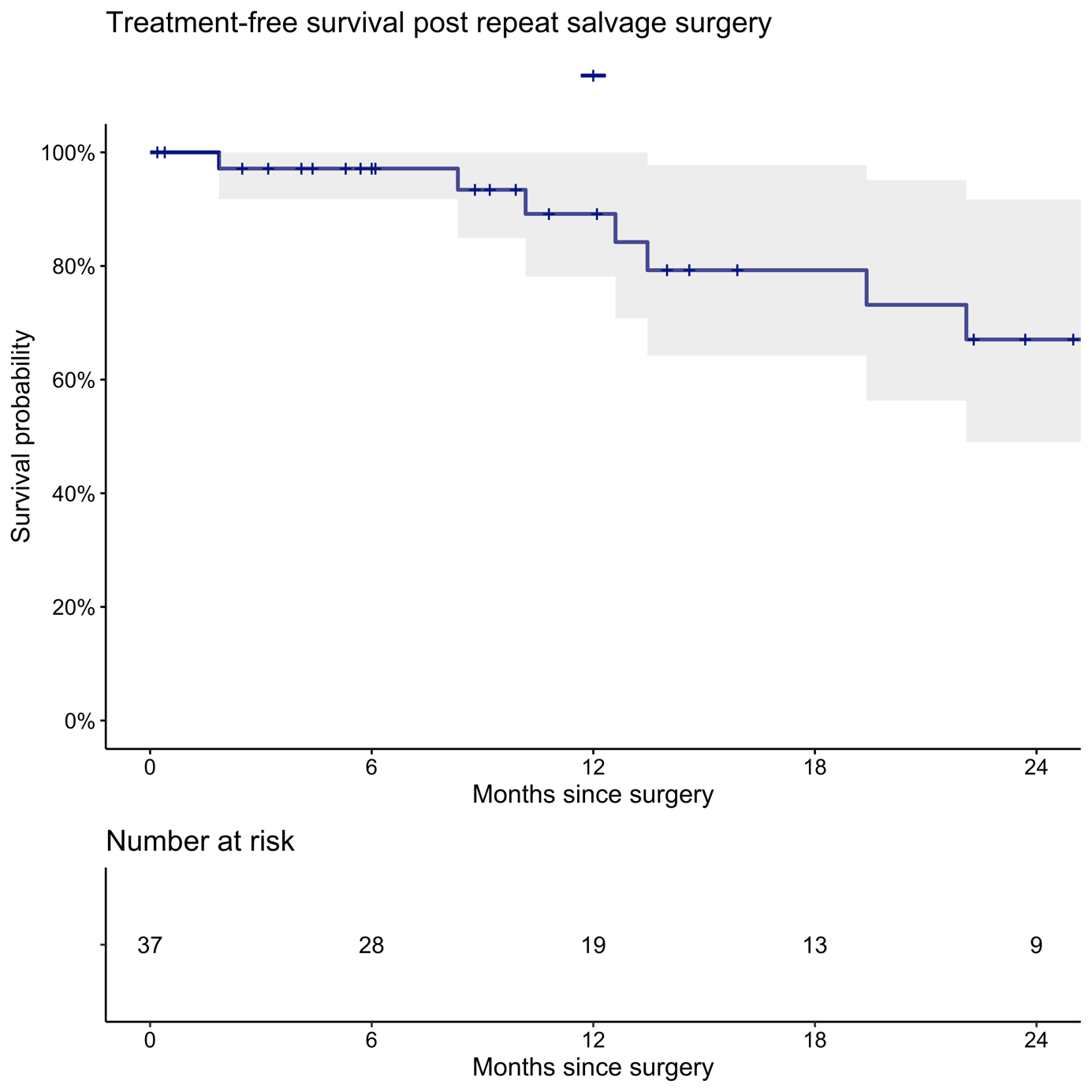


Fig. 3: Kaplan-Meier analyses depicting treatment–free survival rates in patients treated with repeat PSMA–RGS (37 pat.).


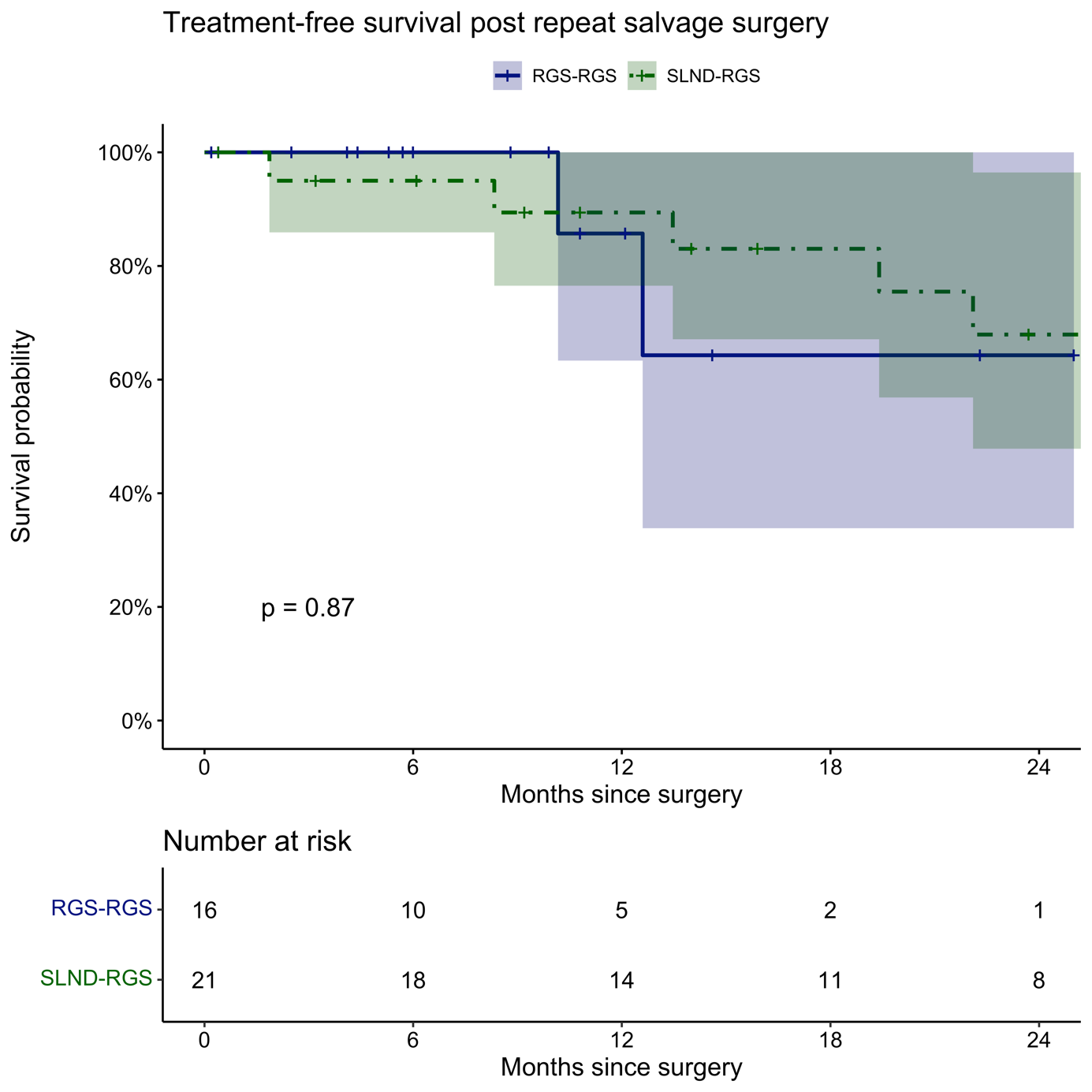


Fig. 4: Kaplan-Meier analyses depicting treatment–free survival rates in patients treated with repeat PSMA–RGS divided by initial treatment (initial SLND in 21 pat. vs. initial PSMA-RGS in 16 pat.).
